# Early life urban exposure as a risk factor for developing obesity and impaired fasting glucose in later adulthood: results from two cohorts in Thailand

**DOI:** 10.1186/s12889-015-2220-5

**Published:** 2015-09-16

**Authors:** Chaisiri Angkurawaranon, Anawat Wisetborisut, Kittipan Rerkasem, Sam-ang Seubsman, Adrian Sleigh, Pat Doyle, Dorothea Nitsch

**Affiliations:** Department of Non-communicable Disease Epidemiology, Faculty of Epidemiology and Population Health, London School of Hygiene and Tropical Medicine, WC1E 7HT London, UK; Department of Family Medicine, Faculty of Medicine, Chiang Mai University, Chiang Mai, Thailand; Department of Surgery, Faculty of Medicine, Chiang Mai University, Chiang Mai, Thailand; Faculty of Human Ecology, Sukhothai Thammathirat Open University, Nonthaburi, Thailand; National Centre for Epidemiology and Population Health, Australian National University, Canberra, ACT Australia

## Abstract

**Background:**

Obesity and obesity related conditions, driven by processes such as urbanization and globalization, are contributing to pronounced cardiovascular morbidity and mortality in developing countries. There is limited evidence on the influence of living in an urban environment in early life on obesity and obesity related conditions later in life in developing countries such as Thailand.

**Methods:**

We used data from two cohort studies conducted in Thailand, the Thai Cohort Study (TCS) and the Chiang Mai University (CMU) Health Worker Study, to investigate the association between early life urban (vs rural) exposure and the later development of obesity. We additionally explored the association between early life urban exposure and impaired fasting glucose in adulthood using data from the CMU Health Worker Study.

**Results:**

Among 48,490 adults from the TCS, 9.1 % developed obesity within 4 years of follow-up. Among 1,804 initially non-obese adults from CMU Health worker study, 13.6 % developed obesity within 5 years of follow-up. Early life urban exposure was associated with increased risk of developing obesity in adulthood in both cohorts. Adjusting for age and sex, those who spent their early lives in urban areas were 1.21 times more likely to develop obesity in the TCS (OR 1.21, 95 % CI 1.12 to 1.31) and 1.65 times more likely in the CMU Health Worker study (OR 1.65, 95 % CI 1.23 to 2.20). These associations remained significant despite adjustment for later life urban exposure and current household income. No evidence for an association was found for impaired fasting glucose.

**Conclusions:**

Early life urban exposure was associated with increased risk of developing obesity in adulthood. These findings support public health intervention programs to prevent obesity starting from early ages.

## Background

The rapidly increasing prevalence of obesity and obesity related conditions such as impaired fasting glucose and diabetes, have been considered a worldwide phenomenon [1]. This is becoming a major issue in developing countries, where obesity and diabetes are now contributing to a pronounced cardiovascular morbidity and mortality [2–4].

Obesity and diabetes are considered to have early life origins [5]. Early life risk factors for childhood and adult obesity include maternal malnutrition, maternal obesity, low birth weight, high birth weight, rapid weight gain in the first year of life, and rapid linear growth in childhood [6–8]. In India, there is evidence that accelerated growth during childhood is linked with obesity, insulin resistance and diabetes later in life [9]. These early life risk factors may also be enhanced or modified by later life environmental influences [10].

Urbanization is linked with many of these early life risk factors [11, 12] and is considered one of the key environmental factors driving obesity and diabetes trends [13]. In developing countries, there is evidence that urbanization may increase the risk of obesity and diabetes through lower physical activity and unhealthy dietary habits such as high glucose consumption [14]. However, urbanization may also be associated with many factors that could decrease the later risk of obesity and diabetes, such as improved socioeconomic status leading to a healthier life style, and better access to care [15]. Compared to developed countries, the rate of urbanization has occurred more rapidly in developing countries [16].

Thailand is a country considered at a tipping point of transition towards becoming a developed country [17]. Obesity has doubled within previous decades [18]. A nationally representative survey in 2009 estimated that around 63.8 % of Thai women and 49.7 % of Thai men aged over 20 were obese (BMI ≥ 25). The prevalence of diabetes in Thailand was around 8.1 and 6.4 %, amongst women and men respectively in 2009 [19]. The study also reported evidence that the prevalence of obesity and diabetes is higher in urban areas. At an ecological level we can thus link urban residence and obesity. A recent cross sectional study among adult healthcare workers in Thailand has also suggested that exposure to urban environments in early life (compared to rural environments) was associated with higher levels of body mass index and fasting glucose in adulthood [20] . However, to properly investigate the influence of early life urban environments on the development of obesity and obesity related conditions, a cohort study is required [21]. Such cohort studies are rare in Thailand. An understanding of the relationship between early life environments with later development of obesity could help identify appropriate targets and timing of interventions to help combat the burden of obesity in Thailand.

We aimed to investigate the association between early life urban exposure with later development of obesity in adulthood using two cohort studies conducted in Thailand between 2005 and 2013. This analysis will also explore whether early life urban exposure remained independently associated with obesity despite later accumulation of adulthood urban exposure. Using data from one of the cohort studies, we will further investigate the association between early life urban exposure with impaired fasting glucose in adulthood.

## Methods

The study utilized data from two cohort studies conducted in Thailand, the Thai Cohort Study (TCS) and the Chiang Mai University (CMU) Health Worker Study.

### The cohort studies

**Fig. 1:**
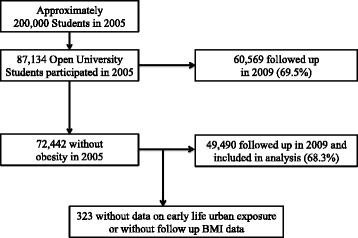
Flow chart of recruitment and follow up in the Thai Cohort Study

The Thai Cohort Study (TCS) is a cohort of students at the Sukhothai Thammathirat Open University in 2005. TCS enrolled 87,142 students residing all over Thailand. The study represented the Thai population well in terms of the median age, gender, geographic and income distribution [22]. This cohort was followed up in 2009 [23]. Using a 20-page questionnaire, data was collected in seven major areas that include socio-demographic details, income and work, food and physical activity, tobacco and alcohol use. This included self-reported body weight and height at baseline and follow up (Fig. 1).

**Fig. 2:**
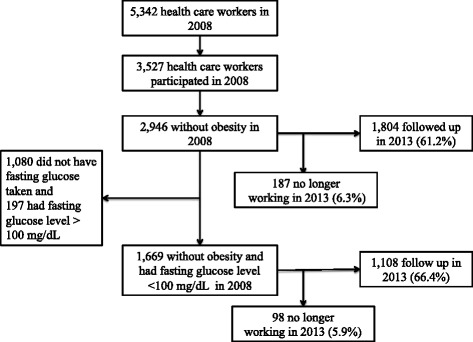
Flow chart of recruitment and follow up in the Chiang Mai University Health Worker Study

The Chiang Mai University (CMU) Health Worker Study surveyed health care workers at the Faculty of Medicine, Chiang Mai University and CMU hospital in 2008. CMU hospital, situated in an urban area of Chiang Mai province, is the largest teaching hospital in Northern Thailand. The study enrolled over 3,500 participants [24]. Self-reported demographic status, monthly income, risk behaviors and common chronic diseases such as hypertension and diabetes were collected using an online questionnaire. Subsequently, all workers were offered a physical examination as well as a complete blood count (CBC) and urine examination according to the Thai National guideline [25]. Other laboratory investigations (fasting glucose and lipid profiles) were only offered to those ages 35 or above. During examinations, standing height and weight were measured using a portable stadiometer and electronic scale. Blood samples were sent to the Central Laboratory Unit in the hospital for processing. The participants were followed up in 2013 [26]. On the day of examinations and laboratory investigations, face-to-face interviews were conducted to obtain a complete migration history from birth to current age (Fig. 2).

### Migration history and urban exposure definition

The TCS used self-classification of urban–rural residence at three life course periods: early life at age 10–12; in 2005 (base line survey); and in 2009 (follow up survey). Participants were asked whether their permanent home during these three periods were considered ‘countryside or city/town’ [27].

The CMU health worker study used an urban classification derived from the Thai urban hierarchy based on population density and the size of municipalities [28]. Urban residence was defined as living in any districts making up Bangkok and Chiang Mai Metropolitan Area. These districts consisted of Muang (Chiang Mai Province), Sarapi, Sanpatong, Hang Dong, Mae Rim, Sansai, Doi Saket, Mae On, Sang Kampang, Muang (Lumphun Province). All other districts in Chiang Mai and Lamphun province were classified as rural. Participants were asked about their entire migration history during their lifetime. District of residence at two life course periods, early life at age 5 and early adulthood at age 20, were determined through interviews.

### Outcome definitions

For obesity, body mass index (BMI) was calculated using body weight (in kilograms) divided by height (in meters) squared. As suggested for Asian populations, obesity was defined as having a body mass index of 25 kg/m^2^ and above [29]. The development of obesity in both cohorts were defined as those who were not obese in the baseline survey and having a BMI of at least 25 kg/m^2^ in the follow up survey.

For impaired glucose and type 2 diabetes, data were only available from the CMU Health Worker study. Preliminary analysis showed that the 5-year incidence for type 2 diabetes (fasting blood glucose of at least 126 mg/dL) was small (<1 %). A fasting glucose of at least 100 mg/dl, which is the criteria for impaired fasting glucose [30], was used as the outcome of interest. The development of impaired fasting glucose was defined as having a fasting glucose of less than 100 mg/dL in 2008 and a fasting glucose level of at least 100 mg/dl in 2013 or taking medication for type 2 diabetes in 2013.

### Other variable of interests

Socioeconomic status, which is considered a key mediator and/or confounder between urbanization and health outcomes [31], was measured through self reported household monthly income. This information was collected during the baseline survey and follow-up survey for both cohorts.

### Analysis strategy

Data from each cohort were analyzed and presented separately. Participants who were pregnant at time of follow up were excluded from the analyses in both cohorts. Demographic data were stratified by gender for descriptive purposes. Demographic factors associated with early life urban residence were tested using chi-square or t-tests. Logistic regression was used to determine the association between early life urban residence and risk of developing obesity and impaired fasting glucose/diabetes. To determine whether early life urban exposure predicts the development of obesity independently of later adulthood urban exposure, further analysis were done adjusting for later urban exposures.

For the TCS, the association between early life urban exposure (at age 10–12) with obesity was further adjusted for urban exposure in 2005 (baseline) and in 2009 (follow up). Additional adjustments were done for current socioeconomic at follow up (in 2009).

For the CMU cohort study, the association between early life urban residence (at age 5) with obesity and impaired fasting glucose/diabetes was further adjusted for early adulthood urban exposure (at age 20). As all health care workers were already working and living in an urban area of Chiang Mai in 2008 (baseline survey), any further adjustments for later life exposure (after 20) would not provide additional information. Additional adjustments were also done for current socioeconomic status at follow up (in 2013). For development of impaired fasting glucose/diabetes, adjustments were also made for family history of type 2 diabetes.

We considered interactions between early life urban exposure and age and sex. In both cohorts, we did not find evidence for interactions between age or sex and early life urban exposure on incident obesity. Thus age and sex were considered as confounders in all final analyses. Potential mediators such as behavioural and lifestyle risk factors, concurrent disease status and current medication were considered to be on the causal pathways between early life urban exposure and development of obesity and impaired fasting glucose. These variables were not adjusted for in all analyses.

### Ethical approval

Informed consent was obtained from all participants in both cohorts. The TCS was approved by the Sukhothai Thammathirat Open University Research and Development Institute (protocol 0522/10) and the Australian National University Human Research Ethics Committee (protocol 2004344). The CMU Health Worker study was approved by Ethical committees from the Faculty of Medicine, CMU (no. 069/2012) and London School of Hygiene and Tropical Medicine (ref. 6521).

## Results

### Follow up rates in TCS

In 2005, 87,134 students agreed to participate (44.0 % response). Using self-reported weight and height, 72,442 were not considered obese. In 2009, 68.3 % (48,490) of non-obese participants were followed up with completed data for analysis (Fig. 1). The participants who were lost to follow up were younger compared to those followed up. However, for gender, baseline BMI, urbanicity of locations in early life and in 2005, there were no obvious differences (Appendix 1).

### Follow up rates in CMU health worker study

In 2008, 3,527 health care workers agreed to participate (66.0 % response). Of these participants, 2,946 were not considered obese. In 2013, 61.2 % (1,804) of non-obese participants were followed up (Fig. 2). The participants who were lost to follow up were more likely to be male and slightly older compared to those who were followed up. However, the baseline BMI and fasting glucose level in 2008 did not differ between those followed up and loss to follow up (Appendix 2).

### Participant characteristics

Demographic characteristics of participants in the TCS are displayed in Table 1, and those of the CMU Health Worker Study in Table 2. The average age of the TCS participants at baseline was 30.7 years (sd = 7.9), which was younger than CMU health worker study participants at baseline (average age 38.3, sd = 8.6). Women represented the majority of both cohorts at 59.0 and 79.9 % for TCS study and the CMU study respectively. For early life urban exposure, 25 % of the TCS participants and over 50 % of the CMU study participants spent their early life living in urban areas. For TCS, the proportion living in urban areas rose from 50.3 % in 2005 to 54.8 % in 2009. By early adulthood (age 20), over 90 % of the participants from the CMU study were living in urban areas.

**Table 1:** Distribution of demographic characteristics and risk of developing obesity among initially non-obese participants of the Thai Cohort Study

Thai cohort study	Female (*N* = 28,635)	Male (*N* = 19,855)	Total (48,490)
Mean age in 2005 (sd)	29.6 (7.4)	32.2 (8.4)	30.7 (7.9)
Monthly household income in 2009: (%:n)			
<10,000 baht	22.1 (6333)	22.5 (4461)	22.3 (10.794)
10,000-20,000 baht	24.6 (7046)	24.7 (4902)	24.6 (11,948)
20,000-50,000 baht	38.4 (10,985)	39.2 (7792)	38.7 (18,777)
>50,000 baht	13.4 (3842)	11.4 (2255)	12.6 (6097)
Missing	1.5 (429)	2,2 (445)	1.8 (874)
Early life location at age 10–12: (%,n)			
Rural	74.6 (21,373)	79.2 (15,731)	76.5 (37,104)
Urban	25.4 (7262)	20.8 (4124)	23.5 (11,386)
Residence in 2005 (%,n)			
Rural	48.0 (13,753)	51.6 (10,235)	49.5 (23,988)
Urban	51.8 (14,8219)	48.2 (9,575)	50.3 (24,394)
Missing	0.2 (63)	0.2 (45)	0.2 (108)
Residence in 2009 (%,n)			
Rural	42.1 (12,059)	46.8 (92,823)	44.0 (21,341)
Urban	56.6 (16,209)	52.2 (10,371)	54.8 (26,580)
Missing	1.3 (367)	1.0 (202)	1.2 (569)
BMI in 2005 (mean, sd)	20.2 (2.1)	21.6 (2.0)	20.8 (2.2)
BMI in 2009 (mean, sd)	21.0 (2.6)	22.4 (2.3)	21.6 (2.6)
Increase in BMI (mean, sd)	0.84 (1.6)	0.75 (1.5)	0.80 (1.6)
Developed obesity (BMI ≥25) by 2009 (%,n)	7.3 (2077)	11.8 (2346)	9.1 (4423)

*BMI* body mass index in kg/m2

**Table 2:** Distribution of demographic characteristics and risk of developing obesity and impaired fasting glucose/diabetes among initially non-obese participants of Chiang Mai University Health Worker Study

CMU health worker study	Female (*N* = 1443)	Male (*N* = 361)	Total (1804)
Mean age in 2008 (sd)	38.4 (8.6)	38.1 (8.3)	38.3 (8.6)
Monthly household income in 2013: (%:n)			
<10,000 baht	7.7 (111)	19.9 (72)	10.2 (183)
10,000-20,000 baht	20.0 (288)	36.3 (131)	23.2 (419)
20,000-50,000 baht	41.4 (598)	27.4 (99)	38.6 (697)
>50,000 baht	30.9 (446)	16.3 (59)	28.0 (505)
Early life location at age 5: (%, n)			
Rural	44.0 (635)	29.1 (105)	41.0 (740)
Urban	56.0 (808)	70.9 (256)	59.0 (1,064)
Early adulthood location at age 20 (%, n)			
Rural	9.8 (141)	7.8 (28)	9.4(169)
Urban	90.0 (1,302)	92.2 (333)	90.6 (1,635)
BMI in 2008 (mean, sd)	21.3 (2.2)	22.8 (2.0)	21.6 (2.2)
BMI in 2013 (mean, sd)	22.7 (2.9)	23.9 (2.5)	22.9 (2.9)
Increase in BMI (mean, sd)	1.38 (1.9)	1.10 (1.8)	1.32 (1.9)
Developed obesity (BMI ≥25) by 2009 (%, n)	12.8 (185)	16.6 (60)	13.6 (245)
Fasting glucose in 2008^a^ (mean, sd)	84.5 (8.9)	87.2 (9.2)	85.0 (9.0)
Fasting glucose in 2013^a^ (mean, sd)	90.1 (8.4)	94.7 (12.6)	91.1 (9.6)
Increase in fasting glucose^a^ (mean, sd)	5.63 (9.9)	7.53 (12.4)	6.03 (10.5)
Developed impaired fasting glucose/diabetes^a^, (%, n)	8.6 (76)	20.2 (45)	10.9 (121)

*BMI* body mass index in kg/m2, Fasting glucose in mg/dL; Impaired fasting glucose/diabetes defined as having fasting glucose ≥ 100 mg/dL

^a^A sample of 885 women and 223 men with fasting glucose measurement

Among the initially non-obese participants in the TCS, the baseline mean body mass index (BMI) in 2005 was 20.2 kg/m^2^ for women and 21.6 kg/m^2^ for men. By 2009, the average increase in BMI for women was 0.84 kg/m^2^ and 0.75 kg/m^2^ for men. The risk of developing obesity was 7.3 in women and 11.8 % in men (Table 1). As participants from the CMU study were older, the baseline BMI and risks of developing obesity were higher than those of the TCS study. Among the initially non-obese participants in the CMU study, the baseline mean body mass index (BMI) in 2008 was 21.3 kg/m^2^ for women and 22.8 kg/m^2^ for men. By 2013, the average increase in BMI for women was 1.38 kg/m^2^ and 1.10 kg/m^2^ for men. The risk of developing obesity over the follow-up period was 12.8 in women and 16.6 % in men (Table 2).

### Distribution of potential confounders

In the TCS, those who lived in an urban areas at age 10–12 were more likely to be older, female and have higher income at follow up in 2009 compared to those who lived in a rural areas at age 10–12. For CMU Health Worker study, those who lived in an urban area at age 5 were more likely to be older, male and have lower income at follow up in 2013 compared to those who spent their early life in rural residences (Table 3).

**Table 3:** Demographic factors, BMI and fasting glucose by early life urban residence

	CMU health worker study			Thai Cohort Study (TCS)		
	Early life residence at age 5			Early life residence at age 10-12		
	Rural *n* = 740	Urban *n* = 1064	*P*-value	Rural *n* = 37,105	Urban *n* = 11,385	*P*-value
Mean age at baseline (sd)	37.2 (8.5)	39.1 (8.5)	<0.01	30.3 (7.7)	31.8 (8.6)	<0.01
Sex: (col %, n)			<0.01			<0.01
Female	85.8	75.9		57.6	63.8.	
Male	14.2	24.1		42.4	36.2	
household income at follow up (col %, n)			<0.01			<0.01
<10,000 baht	6.9	12.4		25.7	11.1	
10,000-20,000 baht	15.1	28.9		26.3	19.4	
20,000-50,000 baht	45.0	34.2		37.3	43.2	
>50,000 baht	33.0	24.5		8,9	24.6	
Missing	0.0	0.0		1.8	1.7	
Mean BMI at baseline (sd)	21.3 (2.2)	21.8 (2.2)	<0.01	20.8 (2.2)	20.8 (2.2)	0.14
Mean BMI at follow up (sd)	22.6 (2.8)	23.2 (2.9)	<0.01	21.5 (2.5)	21.6 (2.6)	<0.01
Mean increase in BMI (sd)	1.34 (1.8)	1.31 (1.9)	0.73	0.80 (1.6)	0.84 (1.6)	0.01
Mean fasting glucose in 2008 (sd)	83.7 (8.5)	84.0 (7.7)	0.53	Not available		
Mean fasting glucose in 2013 (sd)	89.7 (8.8)	90.7 (9.1)	0.07	Not available		
Increase in fasting glucose (sd)	6.0 (9.9)	6.7 (10.0)	0.26	Not available		

BMI at baseline was in 2008 for CMU Health worker study and 2005 for TCS; BMI at follow up was in 2013 for CMU Health Worker study and 2009 for TCS. 32 baht is approximately 1 US dollar; For fasting glucose N for early rural = 429, early urban = 679. Unit for BMI in kg/m^2^, Unit for fasting glucose in mg/dL

### Early life urban exposure as a risk factor for developing obesity

There was consistent evidence from both cohorts that among initially non-obese participants, exposure to urban environments in early life was associated with the later development of obesity in adulthood. Adjusting for age, sex and baseline BMI, those who spent their early life in an urban area were 1.16 times more likely to develop obesity in the TCS (OR 1.16, 95 % CI 1.07 to 1.26) and 1.43 times more likely in the CMU Health Worker study (OR 1.43, 95 % CI 1.01 to 2.02). Adjustment for later adulthood urban residence and current socioeconomic status attenuated these effects. After adjustment for age, sex, later adulthood urban exposure and current socioeconomic status, those spending their early life in an urban area were 1.14 times more likely to develop obesity in the TCS (OR 1.14, 95 % CI 1.04 to 1.24) and 1.44 times more likely in the CMU Health Worker Study (OR 1.46, 95 % CI 0.97 to 2.13) (Table 4). Later adulthood urban exposure in 2009 was also weakly associated with the risk of development of obesity in the TCS (OR 1.10, 95 % CI 1.00 to 1.21) while later adulthood urban exposure at age 20 was not associated with increased risk of developing obesity in the CMU Health Worker study (OR 0.95, 95 % CI 0.49 to 1.82). (Appendix 3).

**Table 4:** Early life urban exposure and risk of developing obesity in adulthood

		Model 1	Model 2	Model 3
	% (n) obese by follow-up	Adjusted OR for obesity (95 % CI) and *p*-value	Adjusted OR for obesity (95 % CI) and *p*-value	Adjusted OR for obesity (95 % CI) and *p*-value
CMU health worker study:				
Early life residence at age 5				
Rural (*n* = 740)	10.3 (76)	Reference	Reference	Reference
Urban (*n* = 1,065)	15.9 (169)	1.43 (1.01 to 2.02) p = 0.04	1.45 (1.00 to 2.22) p = 0.05	1.44 (0.97 to 2.13) p = 0.07
Thai Cohort Study (TCS):				
Early life residence at age 10-12				
Rural (*n* = 37,105)	8.8 (3,251)	Reference	Reference	Reference
Urban (*n* = 11,385)	10.3(1,172)	1.16 (1.07 to 1.26) *p* < 0.01	1.13 (1.04 to 1.23) *p* < 0.01	1.14 (1.04 to 1.24) *p* < 0.01

BMI at baseline was in 2008 for CMU Health worker study and 2005 for TCS; BMI at follow up was in 2013 for CMU Health Worker study and 2009 for TCS. Obesity defined as BMI ≥ 25 kg/m^2^

*Model 1* adjusted odds ratio (OR) for age, sex, baseline BMI

*Model 2* adjusted odds ratio for age, sex, baseline BMI and later urban exposure in adulthood; Results from CMU Health worker study was adjusted for urban residence at age 20, Results from TCS adjusted for urban residence in 2005 and 2009

*Model 3* Adjusted odds ration for age, sex, baseline BMI later urban exposure (same as model 2) and current household income at follow up

No evidence for interactions between early life urban residence and sex in both cohorts

### Early life urban exposure as a risk factor for developing impaired fasting glucose

After adjustment for age, sex and family history of type 2 diabetes, we did not find evidence that exposure to urban environments in early life was associated with development of impaired fasting glucose in the CMU study population (OR 0.91, 0.43 to 1.89). The association between early life urban exposure and development of impaired fasting glucose did not materially alter with additional adjustments for later adulthood urban exposure at age 20 and current socioeconomic status (OR 0.74, 95 % CI 0.31 to 1.78) (Table 5).

**Table 5:** Early life urban exposure and risk of developing impaired fasting glucose/diabetes in adulthood (Fasting Blood glucose ≥ 100 gm/dL)

		Model 1	Model 2	Model 3
CMU health worker study:	% (n) with impaired glucose by follow-up	Adjusted OR for impaired glucose (95 % CI) and *p*-value	Adjusted OR for impaired glucose (95 % CI) and *p*-value	Adjusted OR for impaired glucose (95 % CI) and *p*-value
Early Life Residence at age 5				
Rural (*n* = 429)	10.6 % (45)	Reference	Reference	Reference
Urban (*n* = 679)	11.2 % (76)	0.91(0.43 to 1.90) 0.80	1.01 (0.45 to 2.26) 0.99	0.74 (0.31 to 1.78) 0.50

Fasting glucose at baseline measured in 2008 and followed up was in 2013 in Chiang Mai University (CMU) Health Worker Study

*Model 1* adjusted odds ratio (OR) for age, sex, and family history of diabetes

*Model 2* adjusted odds ratio for age, sex, family history of diabetes and later urban exposure in adulthood at age 20

*Model 3* Adjusted odds ration for age, sex, family history of diabetes, later urban exposure in adulthood at age 20 and current household income at follow up in 2013

No evidence for interactions between early life urban residence and sex

## Discussion

We found consistent evidence from two cohorts that among initially non-obese Thai adults, exposure to an urban environment in early life was associated with increased risk of obesity in adulthood. No evidence was found for an association between early life urban exposure and the development of impaired fasting glucose.

It is important to acknowledge the strengths and limitations of the study before further discussion and interpretations can be made. This study utilized data from two cohort studies which were set up to investigate the role of urbanization and the development of non-communicable diseases (NCDs) in Thailand. Some systematic differences between responders and non-responders were observed but these groups did not differ by baseline BMI and fasting glucose level in the CMU Health worker study, or by baseline BMI and early life urban exposure in the TCS. The definition of urban exposure in both cohorts may be prone to misclassification bias. However, since only limited districts could be considered urban in the CMU Health worker study, urban exposure was unlikely to be misclassified. For TCS, self reported urban classification of residence has been shown to be associated with many aspects of urban living such as higher income, possession of cars and modern household appliances [32]. TCS used self-reported body weight and height to obtain the participant’s BMI, which may also be prone to information bias. However, TCS has conducted a small validation study on these self-reported body weight and height measurements and found that the small discrepancies did not alter any of the associations between health behavior and body mass index [33]. Moreover, using self reported weight and height, the specificity for diagnosis of obesity (BMI ≥ 25) was over 97 % with a positive predictive value of 94 % amongst TCS participants [34]. Although not considered major issues, these imprecise measurements of urban exposures and BMI would be likely to underestimate the associations seen, rather than overestimate them. While we excluded women who were currently pregnant from our analyses, we could not fully exclude the potential effect from postpartum weight retention since we did not record pregnancies between follow up periods. However, given the consistency of the findings in two separate cohorts of different demographics, this was unlikely to be a major issue in our study.

The two cohorts offered different strengths. The cohort composition of TCS suggests that the results are likely to be generalizable to the Thai population [22]. While results from the CMU health worker study may not be generalizable to the Thai population, it offers a unique opportunity to control for some elements of urbanization that may be difficult to disentangle in TCS [26]. The CMU health worker study was restricted to a population with similar access to health services, employment, and similar living and working conditions.

Obesity and diabetes have early life origins that track into adulthood [5]. Urbanization is one of the key drivers linked with childhood obesity [35]. Studies have suggested that BMI in early life is associated with persistently higher BMI in adulthood [36], which in turn is associated with diabetes [37]. The socio-cultural environment associated with urbanization differs between countries, making direct comparison with other settings or populations difficult [38]. However, the results seen in this study are consistent with other studies from developing countries using a life course approach. Lifetime urban exposure was associated obesity and diabetes in Cameroon [13] and increasing BMI and fasting glucose in India [31]. Similar to our study, the associations seen were independent of age, current level of physical activity, and current socioeconomic status and residence.

This study provides evidence that non-obese young adults who had lived in an urban environment in their early life were at increased risk of developing obesity later on in adulthood compared to young adults who had not lived in an urban environment in early life. Although not considered obese at baseline, those with early life urban residence had slightly higher baseline BMI than those without, indicating that the progression to later obesity had already begun. Since most sociocultural and environmental influences associated with urbanization were controlled for in the CMU Health Worker Study, the main mediator for the association is thus likely to be lifestyle influences. Evidence from another study using TCS data suggested that those spending their early lives (at age 10–12) in urban areas were less likely to engaged in regular activity, and more likely to have unhealthy diets in adulthood, than those spending their early life in rural areas [32].

We found no evidence for an association between early life urban exposure and development of impaired fasting glucose in the CMU Health worker study. The risk of developing impaired fasting glucose was low in our study. Moreover, underlying disease conditions may influence other medication use and changes in lifestyles, which can ultimately affect weight gain and glucose homeostasis. Our study was underpowered to detect such associations and potential complexities. A longer duration of follow up and larger sample size is required. However, there are also a number of other plausible reasons why we could not detect an association between early life urban exposure and development of impaired fasting glucose. Since 90 % of participants had been living in an urban area since age 20, there was likely to be a convergence of risks due to similar exposures to social and environmental factors, as seen in India [39]. Any differences due to early life urban exposure could be diluted if these biological risks (such as glucose level) were associated with more current or recent exposures rather than exposure in early life. Unlike BMI, fasting glucose has large biological variability and is more likely to reflect the current glucose homoeostasis [40]. It is also considered to be more susceptible to recent or current lifestyle habits and interventions [41, 42]. In a US study, a three- year history of weight gain among pre-obese adults was found not to result in higher levels of glucose compared to those who had maintain their weight [43]. Another possible explanation why our study could not detect an association between early life urban exposure and development of impaired fasting glucose was that the effect of urban environments may not be consistent across all health outcomes [31]. In some developing countries, including Thailand, while urbanization was associated with higher BMI and blood pressure, it was not always concurrently associated with higher lipid and glucose level [44, 45].

## Conclusions

Consistent evidence from two cohorts found that early life urban exposure was associated with increase risk of developing obesity in adulthood. There are multiple underlying factors driving the association between urbanization and obesity, including maternal conditions, socio-environmental factors, and individual lifestyles [46, 47]. In this study, lifestyle or behavioural factors and access to care are likely to be the key drivers, but further research is needed to understand the factors and mediators underlying the link between early life urban exposure and risk of obesity in Thailand [48]. As already supported by previous research [49], there are benefits to delaying the onset of obesity in order to prevent diabetes and other conditions. Public health intervention programs should be implemented to halt the development of obesity in children and young adults in Thailand.

## Appendix 1

**Table 6:** Demographic characteristics and baseline BMI in responders and non-responders of the Thai Cohort Study (TCS)

		Followed up in 2009 (N=49,813)	Loss follow up in 2009 (N=22,629)
Sex	Female: n(%)	29,371 (59.0)	13,055 (57.8)
	Male: n(%)	20,442 (41.0)	9,573 (42.3)
Age	Mean age in 2005 among female (sd)	29.7 (7.4)	26.2 (6.1)
	Mean age in 2005 years among male (sd)	32.3 (8.5)	28.4 (7.4)
BMI in kg/m^2^	Mean BMI in 2005 among female (sd)	20.2 (2.1)	19.9 (2.0)
	Mean BMI in 2005 among male (sd)	21.6 (2.0)	21.3 (2.0)
Location at age 10-12	Rural	37,757 (76.5)	17,586 (78.6)
	Urban	11,601 (23.5)	4,791 (21.4)
Location in 2005	Rural	24,550 (49.6)	10,649 (47.5)
	Urban	24,934 (50.4)	11,790 (52.5)

## Appendix 2

**Table 7:** Demographic characteristics and baseline BMI and fasting glucose in responders and non-responders of the Chiang Mai University (CMU) Health Worker Study

		Followed up in 2013 (*N*=1,804)	Loss follow up in 2013 (*N*=1,142)
Sex	Female: n(%)	1,441 (79.9)	757 (66.3)
	Male: n(%)	363 (20.1)	385 (33.7)
Age	Mean age in 2008 among female (sd)	38.4 (8.6)	41.4 (9.5)
	Mean age in 2008 years among male (sd)	38.2 (8.4)	42.3 (9.7)
BMI in kg/m^2^	Mean BMI in 2008 among female (sd)	21.3 (2.2)	21.4 (2.3)
	Mean BMI in 2008 among male (sd)	22.8 (2.0)	22.8 (1.9)
Fasting glucose in mg/dL	Mean fasting glucose in 2009 among female* (sd)	84.5 (8.9)	85.3 (9.4)
	Mean fasting glucose in 2008 among male** (sd)	87.1 (9.2)	88.1 (9.9)

Number of female with fasting glucose measurements followed up = 946, lost follow up = 409; number of male with fasting glucose measurement follow up = 249, lost to follow up 189

## Appendix 3

**Table 8:** Early life urban exposure and risk of developing obesity in adulthood

	Model 1	Model 2	Model 3
	Adjusted OR for obesity (95 % CI) and *p*-value	Adjusted OR for obesity (95 % CI) and *p*-value	Adjusted OR for obesity (95 % CI) and *p*-value
CMU Health Worker study:			
Early Life Residence at age 5			
Rural	Reference	Reference	Reference
Urban	1.43 (1.01 to 2.02) *p* = 0.04	1.45 (1.00 to 2.22) *p* = 0.05	1.44 (0.97 to 2.13) *p* = 0.07
Residence at age 20			
Rural	--	Reference	Reference
Urban	--	0.91 (0.48 to 1.74) *p* = 0.79	0.95 (0.49 to 1.82) *p* = 0.87
Thai Cohort Study (TCS):			
Early Life Residence at age 10-12			
Rural	Reference	Reference	Reference
Urban	1.16 (1.13 to 1.30) *p* < 0.01	1.13 (1.12 to 1.31) *p* < 0.01	1.12 (1.09 to 1.28) *p* = 0.01
Residence in 2005			
Rural	--	Reference	Reference
Urban	--	0.99 (0.90 to 1.08) *p* = 0.81	0.98 (0.89 to 1.08) *p* = 0.66
Residence in 2009			
Rural	--	Reference	Reference
Urban	--	1.09 (0.99 to 1.20) *p* = 0.07	1.10 (1.00 to 1.21) *p* = 0.05

BMI at baseline was in 2008 for CMU Health worker study and 2005 for TCS; BMI at follow up was in 2013 for CMU Health Worker study and 2009 for TCS. Obesity defined as BMI ≥ 25 kg/m^2^

*Model 1* adjusted odds ratio (OR) for age, sex, baseline BMI

*Model 2* adjusted odds ratio for age, sex, baseline BMI and later urban exposure in adulthood; Results from CMU Health worker study was adjusted for urban residence at age 20, Results from TCS adjusted for urban residence in 2005 and 2009

*Model 3* Adjusted odds ration for age, sex, baseline BMI later urban exposure (same as model 2) and current household income at follow up
